# Acute transverse myelitis in West Nile Virus, a rare neurological presentation

**DOI:** 10.1016/j.idcr.2021.e01104

**Published:** 2021-03-31

**Authors:** Chinmay Jani, Alexander Walker, Omar Al Omari, Dipesh Patel, Alejandro Heffess, Edward Wolpow, Stephanie Page, Daniel Bourque

**Affiliations:** a Department of Medicine, Mount Auburn Hospital, Cambridge, MA, USA; b Harvard Medical School, Boston, MA, USA; c Department of Radiology, Mount Auburn Hospital, Cambridge, MA, USA; d Division of Neurology, Mount Auburn Hospital, Cambridge, MA, USA; e Division of Infectious Disease, Mount Auburn Hospital, Cambridge, MA, USA

**Keywords:** West Nile Virus, Transverse myelitis, Paralysis

## Abstract

**Introduction:**

West Nile Virus varies in presentation from asymptomatic to a febrile illness often associated with malaise, weakness and maculopapular rash. West Nile neuro-invasive disease often manifests as meningitis, encephalitis, and less commonly acute flaccid paralysis in a "polio-like" presentation. Acute transverse myelitis (ATM) is a rare manifestation. We present a case of neuro-invasive West Nile Virus infection with radiographic evidence of longitudinally extensive transverse myelitis (LETM), a subset of ATM,

**Case narration:**

A 42-year-old male from Massachusetts presented with progressive asymmetric paralysis of 4 days duration after developing a prodrome of fever, neck stiffness and urinary retention. Physical examination demonstrated asymmetric lower extremity weakness Lumbar puncture revealed lymphocytic pleocytosis with normal protein and glucose and a positive West Nile IgM in CSF (4.89, reference <0.90), and West Nile Virus detected by PCR in CSF. His West Nile serum IgM was 3.03 (reference range <0.90) and IgG was <1.30 (reference range <1.30). MRI of the lumbar spine showed findings consistent with the diagnosis of ATM.

**Conclusion:**

With our patient’s presentation of acute onset asymmetrical weakness following a viral illness, we ruled out differentials including demyelinating syndrome such as GBS, inflammation of the meninges through meningitis or meningoencephalitis, or traumatic/ischemic involvement of the spinal cord directly. Due to the MRI findings, his motor weakness and urinary retention, supporting CSF findings and WNV positive serologies, ATM due to WNV infection was the main suspect for his presentation. Although ATM is an uncommon manifestation of WNV, it is imperative to consider this in the differential for patients presenting with acute onset flaccid paralysis in regions where WNV is endemic.

## Highlights

• West Nile Virus can lead to various neurological presentation.
• It is important to identify Acute Transverse Myelitis with the help of imaging.
• It should be in our differentials in patients presenting with muscular weakness in endemic regions.

## Introduction

West Nile Virus (WNV) is an arbovirus from the Flaviviridae family that is commonly found in Africa, Europe, the Middle East, North America and West Asia [1]. WNV infection was first identified in United States during the 1999 outbreak in New-York city when it was found to have transmitted by mosquitos from dead American crows [2]. Currently, it is the most common mosquito borne disease in the United States [3]. From 1999–2018, a total of 50,830 confirmed and probable cases of WNV disease were reported to the CDC from 48 states and the District of Columbia. Since 1999 in Massachusetts, there have been a total of 213 reported cases out of which 164 were neuro-invasive [4].

Manifestations of WNV infection range from asymptomatic infection, WNV fever or a West Nile Neuro-invasive Disease (WNND). In general, the virus is self-limiting, symptoms tend to resolve completely within several weeks. Surveillance serological data in endemic areas suggest that only 20 % of WNV positive patients presented with symptoms, of those only 1% had neurological manifestations [5]. When symptomatic, WNV fever typically presents with fever, chills, fatigue, myalgia, arthralgia, memory impairment, weakness, headache, rash, lymphadenopathy and balance problems [6]. WNND includes severe neurological manifestations of meningitis, encephalitis, and acute flaccid paralysis [7]. Other symptoms like tremor, parkinsonism and myoclonus were also reported [7,]8. Out of 2947 cases of 2003 WNV outbreak of Colorado, 621 neuro-invasive cases were reported including cases having acute respiratory failure secondary to acute diaphragmatic paralysis [9]. Among patients with WNND, roughly half developed WN encephalitis. Additional manifestations include meningitis, acute flaccid paralysis and less commonly, ATM [8,9].

ATM is a heterogeneous focal inflammatory disorder of the spinal cord characterized by acute or subacute development of motor weakness, sensory impairment, and autonomic dysfunction. Etiologies include idiopathic or secondary ATM which can be directly associated with infectious, systemic inflammatory, or multifocal central nervous system disease. Despite the known neuro-invasive nature of WNV the development of ATM is a far less common manifestation. Several cases have been reported including the development of ATM at the initial time of presentation as well as subsequent development of ATM following recovery from acute inflammatory demyelinating polyradiculopathy believed to be precipitated by WNV [10,11]. Various other infectious precipitants including viruses, atypical bacteria as well as parasites have been identified in different cases [12]. In this report, we present a case with radiographic evidence of acute transverse myelitis in the patient with WNV infection.

## Case narration

A 42-year-old male with no previous significant medical history presented to the emergency department with fever, excessive sweating, myalgia, malaise, headache, photophobia, neck stiffness and low back pain. The patient had been on camping trips in Vermont and western Massachusetts, most recently 3 weeks prior to his presentation. Symptoms later manifested as he developed profound weakness in his lower extremities, right side greater than left. He first noted slight difficulty in ambulation, which progressed to lower extremity weakness which prompted him to seek care.

On initial presentation he had a temperature of 101.1 F. Physical examination was notable for normal cognition. He had an unremarkable heart, chest and abdomen exam. On neurologic exam: speech was fluent, cranial nerves II through XII were intact. Bilateral upper extremity strength was 5 out of 5. Right lower extremity strength was 2 out of 5 and left lower extremity strength was 4 out of 5. Sensation was grossly intact. Achilles reflexes were 0–1 + bilaterally, patellar reflex 0 right, left patella 1 + . Gait testing showed him patient to be unstable, having trouble walking. Finger-nose-finger showed no signs of dysmetria or ataxia.

Initial lab work showed normal leukocyte count, mild transaminitis, and lactic acidosis. His inflammatory markers including C-reactive protein and erythrocyte sedimentation rate were normal. Lumbar puncture revealed a lymphocytic pleocytosis with WBC of 155 per mm [3] (0–5) (90 % lymphocytes (<65 %), 8% PMN (0–5 %)), RBC 13 per mm [3] (0–5), protein 56 mg/dL (12–60), glucose 55 mg/dL (40–70). Infectious workup confirmed infection with WNV CSF PCR positive, CSF IGM positive (4.89, reference range < 0.90), positive serum IgM (3.03, <0.90) and, negative serum IgG (within the reference range <1.3). Additional work up was negative for HIV, Herpes simplex viruses, syphilis and Lyme.

**Fig. 1:**
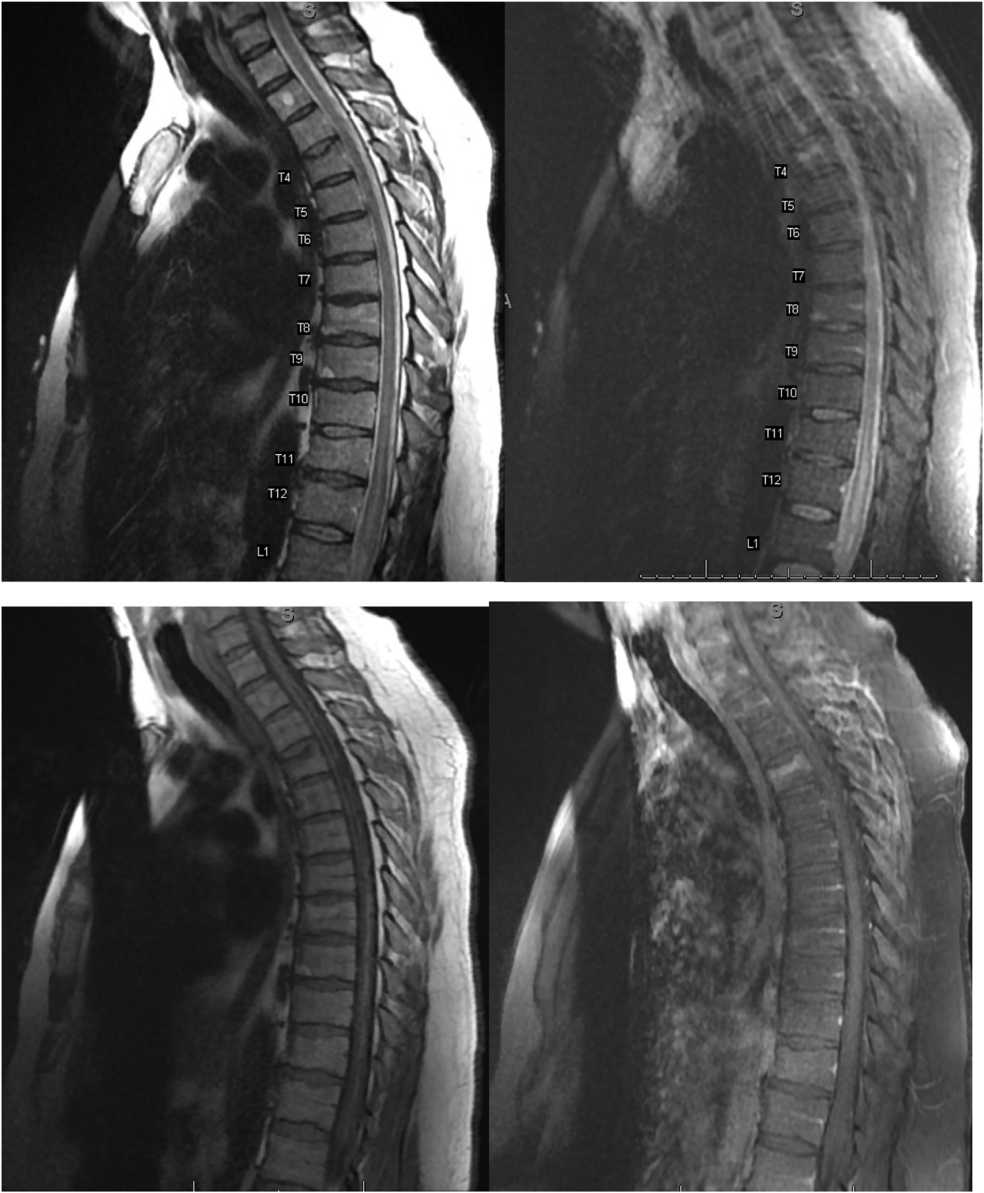
MRI Thoracic spine demonstrating abnormally increased T2 hyperintensity with expansion as seen on Sagittal T2 (top left) and Sagittal STIR (top right) extending from approximately the T10 vertebral level to the conus medullaris which terminates at approximately L1. Given lack of enhancement as demonstrated by the Sagittal T1 (bottom left) and Sagittal T1 post contrast (bottom right), this is most consistent with cord edema. No convincing evidence of cord signal abnormality above these levels. Considering the normal brain MRI, this abnormality most likely represents acute transverse myelitis.

MRI of the lumbar spine demonstrated long segment increased intramedullary T2 signal extending from the conus medullaris to T10 with cord expansion but without enhancement, suggestive of longitudinally extensive transverse myelitis (LETM), a subset of ATM (Fig. 1).

Once a diagnosis of ATM secondary to the WNV had been confirmed the patient was managed symptomatically. A urinary catheter was required for our patient’s acute urinary retention which resolved prior to discharge. He was discharged to an acute rehab facility where his symptoms continued to improve although some degree of weakness was still present at follow-up approximately 1 month later.

## Discussion

We present a rare case of West Nile neuro-invasive disease in which a 42-year-old male with transverse myelitis. He had progressive asymmetric paralysis following several days history of myalgia, headache, malaise, neck stiffness and other symptoms of a viral prodrome. Imaging with MRI of the spine was remarkable for abnormal long segment intramedullary cord edema extending from T10 to the conus medullaris, ending approximately at L1 without associated enhancement.

In addition to the muscular symptoms our patient's presentation of acute urinary retention would support the diagnosis of ATM. Interestingly, our patient lacked the typical sensory level deficit classically depicted in an ATM, or any sensory symptoms, and the presence of purely motor symptoms may favor the acute flaccid myelitis. It is known, however, that the presentation of ATM may vary, sensory, motor and autonomic dysfunction may not always coexist. Additional diagnostic criteria include long segment spinal cord T2 hyperintensity on MR without evidence of cord compression.

Acute flaccid paralysis due to WNV has been used to describe a relatively heterogeneous manifestation of flaccid paralysis with misdiagnosis or underdiagnosis of transverse myelitis. Acute Flaccid paralysis mainly involves anterior spinal segments. It is important to identify as a more unique and a separate manifestation from acute flaccid paralysis. With regards to imaging, the ability to discern between these variants of myelitis is not always possible. The presence of sole involvement of the anterior motor cells would favor flaccid myelitis, however, predominant involvement of the anterior horn has been seen with transverse myelitis [10]. Even though MRI is the mainstay of neuroimaging for WNV, it is relatively insensitive with only 20%–70% of patients with acute WNND reporting abnormalities. The classic spine findings of transverse myelitis is long segment hyperintensity on T2-weighted and fluid attenuated inversion recovery (FLAIR) images which is isointense on T1-weighted images (T1WI) without enhancement [7]. Although the enhancement pattern can be variable. There have been reports of development of FLAIR/T2 hyperintensity in Thalamus, parathalamic region, cerebellum, hippocampi-parahippocampi as well as Substantia Nigra [13,14]. Although not many reports have documented spinal imaging findings, Ali et al. reported 3 different cases with abnormal spinal findings out of which one - had enhancements of the cauda equina and lumbosacral nerve routes on contrast enhanced T1W1 imaging. In the second patient, spinal T2WI (T2-weighted Image) revealed diffusely increased intensity in conus medullaris and thoracic spinal cord whereas Gadolinium-enhanced T1WI showed enhancement in cauda-equina, conus medullaris. In third patient, T2WI of the spine showed patchy increased signal intensity in the thoracic spinal cord, with enhancement in these areas on contrast-enhanced T1WI [15]. In our patient, MRI showed long segment increased intramedullary T2 signal extending from the conus medullaris to T10 with cord expansion but without enhancement. Similar findings with enhancements in C3-C7 region were seen in the report by Martinovic er al [10]. Further, EMG studies demonstrating classic findings for lower motor neuron involvement with fibrillations and extended duration/polyphasic motor unit action potentials are helpful in differentiating between demyelinating disorders like GBS but may not be able to delineate between various forms of myelitis. Although guidelines vary, therapeutic interventions with steroids / IVIG have been shown to have some positive outcomes in ATM [16]. However, it is unclear if there is benefit in the setting of an active viral infection leading to ATM. In our patient, we did not give steroids due to lack of conclusive supporting evidence. Patient showed improvement with symptomatic management alone.

There is wide array of viruses known to cause viral myelopathy with varying presentations based on the individual virus’s predilection towards different areas of gray and white matter within the spinal cord as well as varying spinal cord heights. HIV for example is known to cause white matter destruction of the dorsal columns and corticospinal tracts most commonly in the thoracic spinal cord. This typically presents with lower extremity weakness, spastic paraparesis and sensory level defect. Similarly, HTLV-1 may cause lower extremity weakness, spastic paresis and hyperreflexia secondary to involvement of lateral corticospinal tracts, typically also associated with early bladder involvement. Herpes viruses exhibit a propensity for spinal cord involvement with presentations varying on their location of the respective dormant dorsal root ganglions. HSV-2, for example, can have retrograde migration of the cauda equina to the conus and lower spinal cord with symptoms varying from pain and paresthesia to urinary retention and sensory defects. Further, retrograde migration of VZV is known to precipitate myelitis, oftentimes localized to a similar segment as the correlating vesicular rash. It is well-known the poliovirus has a predilection for anterior horn involvement leading to acute flaccid paralysis with an absence of sensory defects. West Nile Viruses and other Flaviviruses have been shown to manifest in similar fashion with cases of ATM described in Dengue and Japanese encephalitis [17]. Currently, it is still not clear whether the presence of active viral infection or the patient's autoimmune response is the driving factor for ATM secondary to viral disease [18]. However, the presence of greater than 3 spinal cord level involvement favors longitudinally extensive transverse myelitis secondary to WNV is a rare manifestation that is not well described [10].

## Conclusion

West Nile Virus, although known to cause meningitis, encephalitis and acute flaccid paralysis, was found to precipitate longitudinally extensive transverse myelitis, subset of acute transverse myelitis as demonstrated by radiographic evidence displaying enhancement in 3 contiguous spinal cord segments as well as CSF analysis consistent with WNND. This case adds another rare possible neuro-invasive presentation to the wide variety of heterogeneous neurological diseases of WNV infection.

## Author contributions

Each of the authors significantly contributed to this manuscript. Dr Jani, Dr Walker, Dr Wolpow, Dr Page and Dr Bourque made significant contributions to the concept and design of this paper. Dr Jani, Dr Al Omari, Dr Walker, Dr Patel, Dr Heffes, Dr Wolpow, Dr Page and Dr Bourque were greatly involved in drafting manuscript. Dr Page, Dr Wolpow and Dr Bourque were involved in critical revision of the manuscript and contributed vital intellectual content. Chinmay Jani (corresponding author, guarantor) takes responsibility for the content of the manuscript, including its data.

## Funding

This research did not receive any specific grant from funding agencies in the public, commercial, or not-for-profit sectors.

## Consent

Written informed consent was obtained from the patient for publication of this case report and accompanying images. A copy of the written consent is available for review by the Editor-in-Chief of this journal on request.

## Ethical approval

Informed Consent was obtained for the case report.

## Declaration of Competing Interest

The authors report no declarations of interest.

## References

[1] WHO Fact sheets of West Nile Virus. https://www.who.int/news-room/fact-sheets/detail/west-nile-virus Accessed on 11/27/2020.

[2] Nash D., Mostashari F., Fine A., Miller J., O’Leary D., Murray K. (2001). The outbreak of West Nile virus infection in the New York City area in 1999. N Engl J Med.

[3] Carson P.J., Konewko P., Wold K.S., Mariani P., Goli S., Bergloff P. (2006). Long-term clinical and neuropsychological outcomes of West Nile virus infection. Clin Infect Dis.

[4] (2018). West Nile virus: final cumulative maps & data for 1999–2018 [Internet]. https://www.cdc.gov/westnile/statsmaps/cumMapsData.html.

[5] Fratkin J.D., Leis A.A., Stokic D.S., Slavinski S.A., Geiss R.W. (2004). Spinal cord neuropathology in human West Nile virus infection. Arch Pathol Lab Med.

[6] Patnaik J.L., Harmon H., Vogt R.L. (2006). Follow-up of 2003 human West Nile virus infections, Denver, Colorado. Emerg Infect Dis.

[7] Davis L.E., DeBiasi R., Goade D.E., Haaland K.Y., Harrington J.A., Harnar J.B. (2006). West Nile virus neuroinvasive disease. Ann Neurol.

[8] Sejvar J.J., Haddad M.B., Tierney B.C., Campbell G.L., Marfin A.A., Van Gerpen J.A. (2003). Neurologic manifestations and outcome of West Nile virus infection. JAMA.

[9] Sejvar J.J., Bode A.V., Marfin A.A., Campbell G.L., Ewing D., Mazowiecki M. (2005). West Nile virus-associated flaccid paralysis. Emerg Infect Dis.

[10] Martinovic V., Kisic-Tepavcevic D., Kacar A., Mesaros S., Pekmezovic T., Drulovic J. (2019). Longitudinally extensive transverse myelitis in a patient infected with West Nile virus. Mult Scler Relat Disord.

[11] Johal J., Apolo R.C., Ravichandran A., Sivakumar K., Varade S., Walsh A. (2020). Acute Inflammatory Demyelinating Polyradiculoneuropathy (AIDP) and transverse myelitis: a tale of West Nile Virus infection (5106). Neurology.

[12] Kibiki G.S., Murphy D.K. (2006). Transverse myelitis due to trypanosomiasis in a middle aged Tanzanian man. J Neurol Neurosurg Psychiatry.

[13] Bosanko C.M., Gilroy J., Wang A.M., Sanders W., Dulai M., Wilson J. (2003). West Nile virus encephalitis involving the substantia nigra: neuroimaging and pathologic findings with literature review. Arch Neurol.

[14] Szatmary G., Leis A.A. (2015). Concurrent West Nile virus infection in pneumococcal meningitis: clinical and MRI features. J Neuroimaging.

[15] Ali M., Safriel Y., Sohi J., Llave A., Weathers S. (2005). West Nile virus infection: MR imaging findings in the nervous system. AJNR Am J Neuroradiol.

[16] Krishnan C., Kaplin A.I., Pardo C.A., Kerr D.A., Keswani S.C. (2006). Demyelinating disorders: update on transverse myelitis. Curr Neurol Neurosci Rep.

[17] Lyons J.L. (2015). Myelopathy associated with microorganisms. Continuum (Minneap Minn).

[18] Jeffery D.R., Mandler R.N., Davis L.E. (1993). Transverse myelitis: retrospective analysis of 33 cases, with differentiation of cases associated with multiple sclerosis and parainfectious events. Arch Neurol.

